# Crystal structure and Hirshfeld surface analysis of (3a*R*,4*S*,7*S*,7a*S*)-4,5,6,7,8,8-hexa­chloro-2-{6-[(3a*R*,4*R*,7*R*,7a*S*)-4,5,6,7,8,8-hexa­chloro-1,3-dioxo-1,3,3a,4,7,7a-hexa­hydro-2*H*-4,7-methano­isoindol-2-yl]hex­yl}-3a,4,7,7a-tetra­hydro-1*H*-4,7-methano­iso­indole-1,3(2*H*)-dione

**DOI:** 10.1107/S2056989021006952

**Published:** 2021-07-09

**Authors:** Aygun I. Alikhanova, Zeliha Atioğlu, Mehmet Akkurt, Sixberth Mlowe

**Affiliations:** a Institute of Polymer Materials, National Academy of Sciences of Azerbaijan, Sumgayit, 5004, Azerbaijan; b Department of Aircraft Electrics and Electronics, School of Applied Sciences, Cappadocia University, Mustafapaşa, 50420 Ürgüp, Nevşehir, Turkey; c Department of Physics, Faculty of Sciences, Erciyes University, 38039 Kayseri, Turkey; d University of Dar es Salaam, Dar es Salaam University College of Education, Department of Chemistry, PO Box 2329, Dar es Salaam, Tanzania

**Keywords:** crystal structure, pyrrolidine ring, cyclo­pentane ring, cyclo­hexane ring, Hirshfeld surface analysis

## Abstract

The mol­ecule of the title compound, C_24_H_16_Cl_12_N_2_O_4_, is generated by a crystallographic inversion centre at the midpoint of the central C—C bond. A kink in the mol­ecule is defined by a torsion angle of −169.86 (15)° about this central bond of the alkyl bridge. The pyrrolidine ring is essentially planar [max. deviation = 0.014 (1) Å]. The cyclo­hexane ring has a boat conformation, while both cyclo­pentane rings adopt an envelope conformation. In the crystal structure, mol­ecules are linked by inter­molecular C—H⋯O, C—H⋯Cl and C—Cl⋯π inter­actions, and short inter­molecular Cl⋯O and Cl⋯Cl contacts, forming a three-dimensional network.

## Abstract 2

The asymmetric unit contains one-half of the formula unit of the title compound. The crystal structure is stabilized by inter­molecular C—H⋯O, C—H⋯Cl and C—Cl⋯π inter­actions, and short inter­molecular Cl⋯O and Cl⋯Cl contacts, forming a three-dimensional network.

## Chemical context   

*N*-heterocyclic compounds are of inter­est in the fields of synthetic organic chemistry, coordination chemistry and medicinal chemistry because of their important biological properties (Mahmoudi *et al.*, 2016 ▸, 2017*a* ▸,*b* ▸,*c* ▸, 2018*a* ▸,*b* ▸; 2019 ▸; Viswanathan *et al.*, 2019 ▸). For this reason, many approaches have been developed for their efficient and versatile synthesis (Gurbanov *et al.*, 2017 ▸, 2018*a* ▸,*b* ▸; Ma *et al.*, 2017*a* ▸,*b* ▸). On the other hand, *N*-heterocycles or *N-*ligands can also be used as precursors in the synthesis of coordination compounds (Ma *et al.*, 2020 ▸, 2021 ▸; Mahmudov *et al.*, 2013 ▸), and as building blocks in the construction of supra­molecular structures as they have both hydrogen-bond donor and acceptor capabilities (Gurbanov *et al.*, 2020*a* ▸; Kopylovich *et al.*, 2011*a* ▸,*b* ▸; Asgarova *et al.*, 2019 ▸). In fact, attachment of suitable functional groups to *N*-ligands can improve their solubility and the catalytic activity of the corresponding coordination compounds (Mizar *et al.*, 2012 ▸; Gurbanov *et al.*, 2020*b* ▸; Khalilov *et al.*, 2011 ▸, 2018*a* ▸,*b* ▸; Maharramov *et al.*, 2019 ▸; Shikhaliyev *et al.*, 2019 ▸; Shixaliyev *et al.*, 2014 ▸). Inter­molecular halogen bonds and other types of non-covalent inter­actions in halogenated *N*-heterocyclic compounds can improve their solubility and other functional properties. In order to continue our work in this perspective, we have synthesized a new halogenated *N*-heterocyclic compound, (3a*R*,4*S*,7*S*,7a*S*)-4,5,6,7,8,8-hexa­chloro-2-{6-[(3a*R*,4*R*,7*R*,7a*S*)-4,5,6,7,8,8-hexa­chloro-1,3-dioxo-1,3,3a,4,7,7a-hexa­hydro-2*H*-4,7-methano­isoindol-2-yl]hex­yl}-3a,4,7,7a-tetra­hydro-1*H*-4,7-methano­iso­indole-1,3(2*H*)-dione, which provides multiple iner­molecular non-covalent inter­actions. 
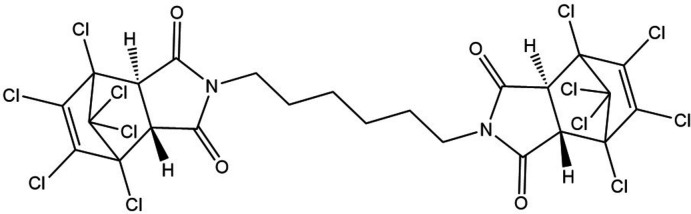


## Structural commentary   

**Figure 1:**
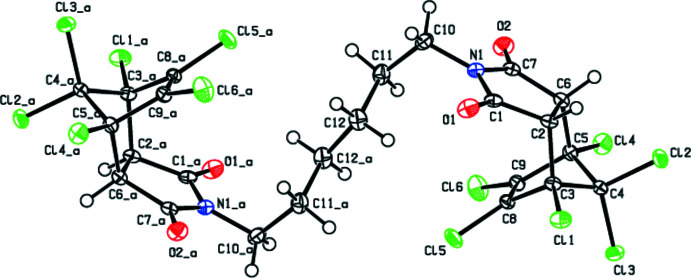
The mol­ecular structure of the title compound with displacement ellipsoids for the non-hydrogen atoms drawn at the 50% probability level. [Symmetry code: (*a*) 2 − *x*, 1 − *y*, −*z*].

The mol­ecule of the title compound is generated by a crystallographic inversion centre at the midpoint of the central C—C bond. A kink in the mol­ecule is defined by the C10—C11–C12—C12_a torsion angle of −169.86 (15)° about this central bond of the alkyl bridge (Fig. 1 ▸). The pyrrolidine ring (N1/C1/C2/C6/C7) is essentially planar [maximum deviation = −0.014 (1) Å for N1]. The cyclo­hexane ring (C2/C3/C5/C6/C8/C9) has a boat conformation [the puckering parameters (Cremer and Pople, 1975 ▸) are *Q*_T_ = 0.9300 (14) Å, θ = 89.99 (9)°, φ = 59.37 (9)°], while both the cyclo­pentane rings (C2–C6 and C3–C5/C8/C9) adopt an envelope conformation [*Q*(2) = 0.6308 (14) Å, φ(2) = 252.44 (13)° and *Q*(2) = 0.5835 (14) Å, φ(2) = 215.53 (14)°, respectively] with the C4 atom bearing the di­chloro­methane group as the flap.

## Supra­molecular features and Hirshfeld surface analysis   

**Figure 2:**
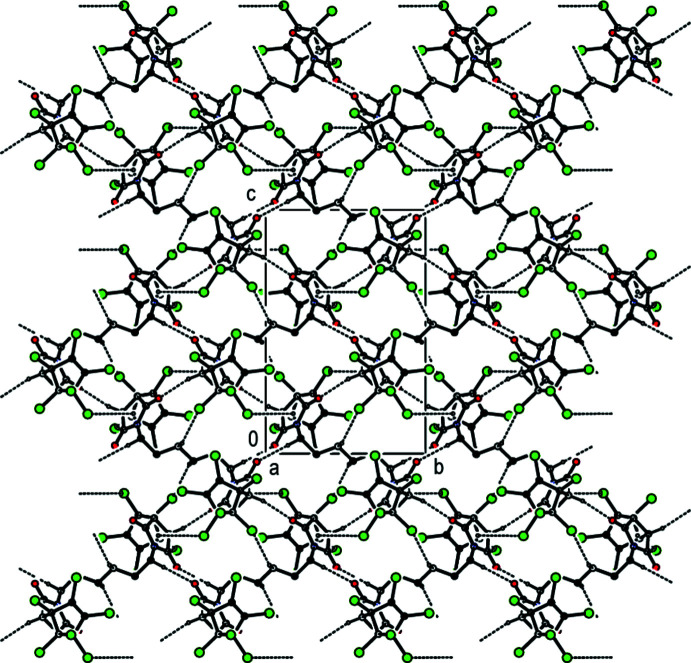
Crystal packing of the title compound viewed along the *a*-axis direction. C—H⋯O, C—H⋯Cl hydrogen bonds and C—Cl⋯π inter­actions (Table 1 ▸) are represented by dashed lines. H atoms not involved in hydrogen bonding are omitted for clarity.

In the crystal structure, mol­ecules are linked by inter­molecular C—H⋯O, C—H⋯Cl and C—Cl⋯π inter­actions (Table 1 ▸), and short inter­molecular contacts, listed in Table 2 ▸, forming a three-dimensional network (Figs. 2 ▸ and 3 ▸).

**Figure 4:**
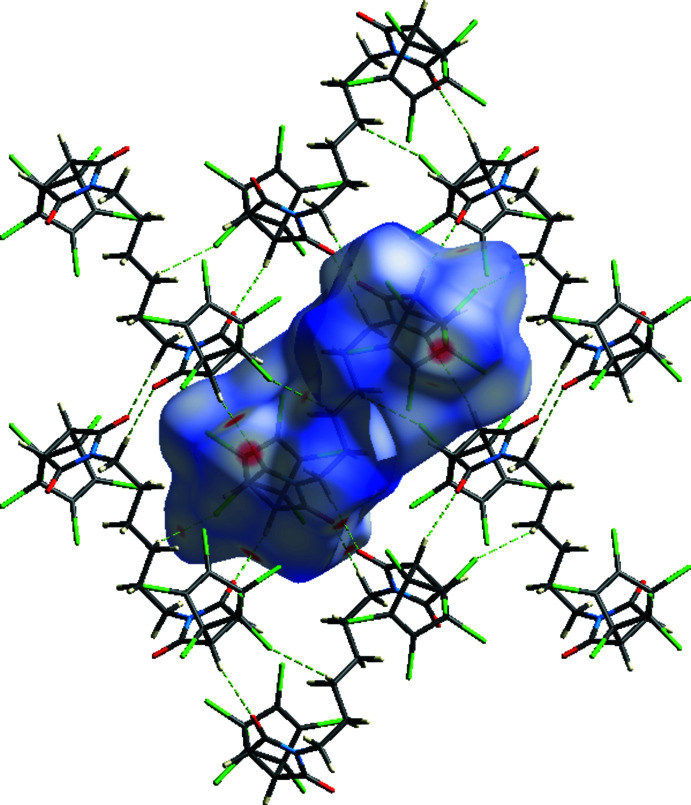
A view of the Hirshfeld surface for the title compound, plotted over *d*_norm_ in the range −0.1922 to 1.7149 a.u. together with inter­acting neighbouring mol­ecules.

**Figure 5:**
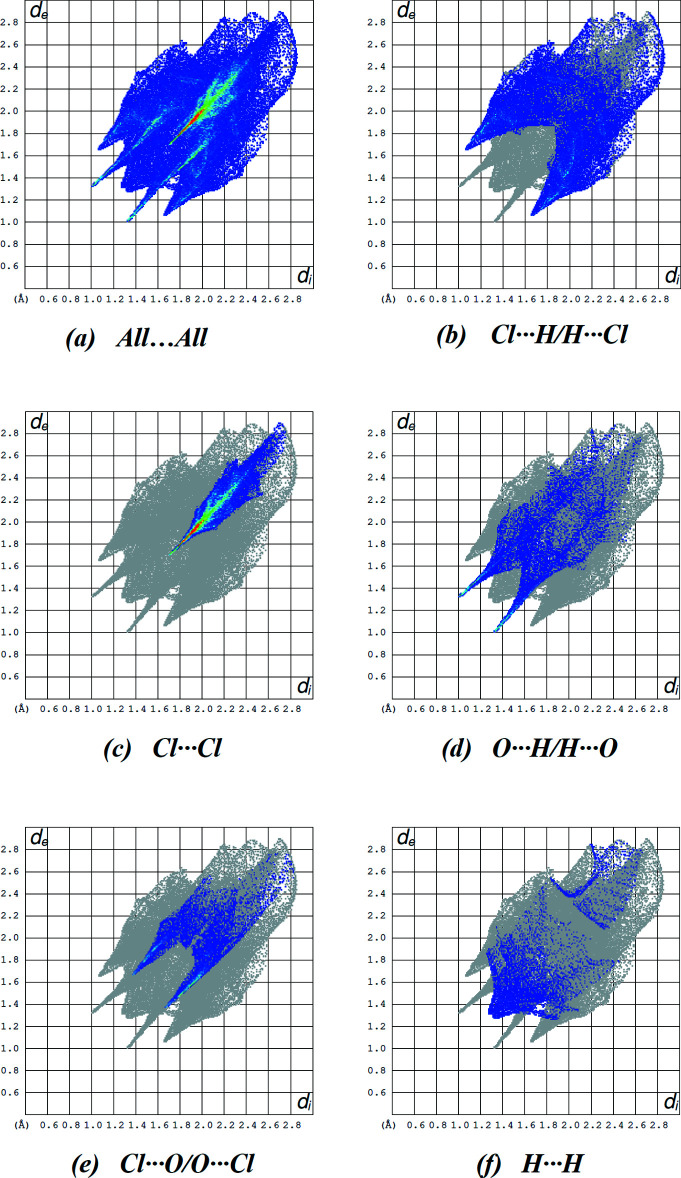
A view of the two-dimensional fingerprint plots for the title compound, showing (*a*) all inter­actions, and delineated into (*b*) Cl⋯H/H⋯Cl, (*c*) Cl⋯Cl and (*d*) O⋯H/H⋯O, (*e*) Cl⋯O/O⋯Cl and (*f*) H⋯H inter­actions. The *d*_i_ and *d*_e_ values are the closest inter­nal and external distances (in Å) from given points on the Hirshfeld surface.

In order to visualize the inter­molecular inter­actions (Table 2 ▸) in the crystal of the title compound, a Hirshfeld surface analysis was carried out using *Crystal Explorer 17.5* (Turner *et al.*, 2017 ▸). Fig. 4 ▸ shows the Hirshfeld surface plotted over *d*_norm_ in the range −0.1922 to 1.7149 a.u. The red spots on the Hirshfeld surface represent C—H⋯O and C—H⋯Cl contacts. Fig. 5 ▸ shows the full two-dimensional fingerprint plot and those delineated into the major contacts: Cl⋯H/H⋯Cl (33.6%; Fig. 5 ▸ *b*), Cl⋯Cl (29.3%; Fig. 5 ▸ *c*), O⋯H/H⋯O (13.9%; Fig. 5 ▸ *d*), Cl⋯O/O⋯Cl (11.4%; Fig. 5 ▸ *e*) and H⋯H (7.0%; Fig. 5 ▸ *f*) inter­actions. The remaining other weak inter­actions (contribution percentages) are Cl⋯C/C⋯Cl (3.2%), Cl⋯N/N⋯Cl (1.4%) and C⋯H/H⋯C (0.2%).

## Database survey   

Four related compounds containing the methano­iso­indole moiety were found in the Cambridge Structural Database (CSD, version 5.42, update of November 2020; Groom *et al.*, 2016 ▸): 4,5,6,7,8,8-hexa­chloro-2-[2-(3,4-di­meth­oxy­phen­yl)eth­yl]-3a,4,7,7a-tetra­hydro-1*H*-4,7-methano­iso­indole-1,3(2*H*)-dione (refcode COHTUR: Manohar *et al.*, 2019 ▸), 5-hy­droxy-4-(4-methyl­phen­yl)-4-aza­tri­cyclo­[5.2.1.0^2,6^]dec-8-en-3-one (QOVCAH: Aslantaş *et al.*, 2015 ▸), (3a*R*,4*S*,7*R*,7a*S*)-2-(perfluoro­pyridin-4-yl)-3a,4,7,7a-tetra­hydro-1*H*-4,7-methano­iso­indole-1,3(2*H*)-dione (MOJFUP: Peloquin *et al.*, 2019 ▸) and (3a*R*,4*S*,7*R*,7a*S*)-2-[(perfluoro­pyridin-4-yl)­oxy]-3a,4,7,7a-tetra­hydro-1*H*-4,7-methano­iso­indole-1,3(2*H*)-dione (MOJ­GAW: Peloquin *et al.*, 2019 ▸).

In COHTUR, the six-membered ring of the norbornene moiety adopts a boat conformation and the two five-membered rings have envelope conformations. The pyrrolidine ring makes a dihedral angle of 14.83 (12)° with the 3,4-di­meth­oxy­phenyl ring, which are attached to each other by an extended N—CH_2_—CH_2_—C_ar_ bridge. In the crystal of COHTUR, weak C—H⋯O hydrogen bonds link the mol­ecules, forming a cyclic 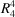 (48) ring motif (Bernstein *et al.*, 1995 ▸). The mol­ecules are stacked in layers held together by offset π–π inter­actions, with a centroid–centroid distance of 3.564 (1) Å for the pyrrolidine and benzene rings. There is also an inter­molecular C—Cl⋯π inter­action present.

In the crystal of QOVCAH, the cyclo­hexene ring adopts a boat conformation, and the five-membered rings have envelope conformations with the bridging atom as the flap. Their mean planes are oriented at a dihedral angle of 86.51 (7)°. The mol­ecular structure is stabilized by a short intra­molecular C—H⋯O contact. In the crystal, mol­ecules are linked by O—H⋯O hydrogen bonds, forming chains propagating along [100]. The chains are linked by C—H⋯π inter­actions, forming slabs parallel to (001).

The compound MOJFUP crystallizes in the triclinic space group *P* 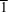 with two mol­ecules, *A* and *B*, in the asymmetric unit, and MOJGAW in the monoclinic space group *P*2_1_/*n* with one mol­ecule per asymmetric unit. The synthesis of both compounds is conducted using *endo* starting materials, and the same configuration is observed in the resulting crystal structures. In MOJFUP, steric inter­actions between the *ortho*-fluorine atoms and the carbonyl oxygen atoms prevents free rotation about the nitro­gen–*ipso*-carbon bond, which is evidenced by separate ^19^F NMR peaks in solution for the *ortho*-F atoms. In mol­ecule *A*, the 2,3,5,6-tetra­fluoro­pyridine plane is rotated by 58.05 (5)° relative to the pyrrolidine plane and the corresponding dihedral angle for mol­ecule *B* is 61.65 (7)°. The addition of an oxygen atom between N and C in the bridge between the ring systems in MOJGAW alleviates this steric restriction and only one ^19^F NMR peak in solution is observed for the *ortho*-F atoms; even so, the dihedral angle between the 2,3,5,6-tetra­fluoro­pyridine and pyrrolidine planes in the crystal of MOJGAW of 84.01 (5)° is larger than that found in MOJFUP.

The main directional inter­actions in the crystal structures of MOJFUP and MOJGAW are of the type C—H⋯O, C—H⋯F, C—O⋯π, and C—F⋯π. In both compounds, weak hydrogen-bonding inter­actions are observed for the hydrogen atom(s) α to the carbonyl groups (C—H⋯O and C— H⋯F in MOJFUP; C—H⋯O in MOJGAW) and the olefinic hydrogen atoms (C—H⋯F in MOJFUP; C—H⋯O in MOJGAW). A weak inter­action is also observed for a bridge hydrogen atom in MOJGAW, C—H⋯F. The packing is further aided by π-inter­actions with the pyridine ring in MOJGAW.

## Synthesis and crystallization   

To 741 mg (2 mmol) of (3a*R*,4*R*,7*R*,7a*S*)-4,5,6,7,8,8-hexa­chloro-3a,4,7,7a-tetra­hydro-4,7-methano­isobenzo­furan-1,3-dione were added 0.12 mL (1 mmol) of hexane-1,6-di­amine and 25 mL of di­methyl­formamide, and the mixture was stirred for 6 h at 373 K. Then, the reaction mixture was cooled to room temperature and poured into cold water. The obtained precipitate was filtered off, washed with water, recrystallized from chloro­form and dried under vacuum. Yellow powder, yield 92%, m.p 404–405 K (decomp.). Analysis calculated for C_24_H_16_Cl_12_N_2_O_4_ (*M*_r_ = 821.80): C 35.08, H 1.96, N 3.41%; found: C 35.03, H 2.00, N 3.35%. ESI–MS: *m*/*z*: 822.9 [*M*_r_ + H]^+. 1^H NMR (300.130 MHz) in acetone-*d*_6_, inter­nal TMS, δ (ppm): 1.29–3.43 (12H, 6CH_2_), 3.86 (4H, CH). ^13^C{^1^H} NMR (75.468 MHz, acetone-*d*_6_). δ: 25.8 (2CH_2_), 27.2 (2CH_2_), 39.3 (4C–H), 52.0 (2CH_2_), 79.3 (4CCl), 104.4 (2CCl_2_), 130.9 (2ClC=CCl) and 170.2 (4C=O). Off-white prismatic crystals suitable for X-ray analysis were obtained by slow evaporation of a chloro­form–hexane (1/1, *v*/*v*) mixture.

## Refinement   

Crystal data, data collection and structure refinement details are summarized in Table 3 ▸. All C-bound H atoms were positioned geometrically and refined using a riding model, with C—H = 0.99 (methyl­ene) and 1.00 Å (methine), with *U*_iso_(H) = 1.2*U*_eq_(C). Two reflections (100 and 002), affected by the incident beam-stop, and owing to poor agreement between observed and calculated intensities, two outliers (136 and 118) were omitted in the final cycles of refinement. 

## Supplementary Material

Crystal structure: contains datablock(s) I. DOI: 10.1107/S2056989021006952/vm2251sup1.cif

Structure factors: contains datablock(s) I. DOI: 10.1107/S2056989021006952/vm2251Isup2.hkl

CCDC reference: 2094787

Additional supporting information:  crystallographic information; 3D view; checkCIF report

## Figures and Tables

**Figure 3:**
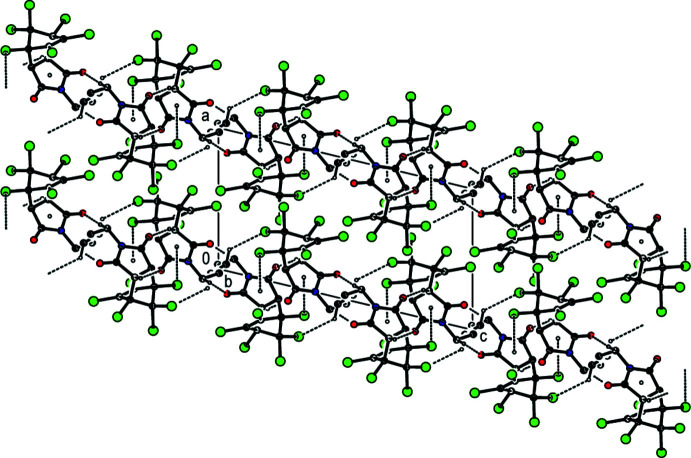
Crystal packing viewed along the *b* axis, with inter­molecular inter­actions shown as in Fig. 2 ▸. H atoms not involved in hydrogen bonding are omitted for clarity.

**Table 1:** Hydrogen-bond geometry (Å, °) *Cg*1 is the centroid of the N1/C1/C2/C6/C7 pyrrolidine ring.

*D*—H⋯*A*	*D*—H	H⋯*A*	*D*⋯*A*	*D*—H⋯*A*
C6—H6⋯O1^i^	1.00	2.43	3.3867 (16)	161
C10—H10*A*⋯O2^ii^	0.99	2.45	3.4402 (17)	178
C12—H12*B*⋯Cl2^iii^	0.99	2.80	3.5299 (15)	131
C3—Cl1⋯*Cg*1^iii^	1.75 (1)	3.89 (1)	4.9389 (14)	117 (1)

Symmetry codes: (i) -x+2, y-{\script{1\over 2}}, -z+{\script{1\over 2}}; (ii) -x+2, -y, -z; (iii) -x+2, y+{\script{1\over 2}}, -z+{\script{1\over 2}}.

**Table 2:** Summary of short inter­atomic contacts (Å) in the title compound

Contact	Distance	Symmetry operation
Cl3⋯Cl2	3.4333 (5)	1 − *x*, {1\over 2} + *y*, {1\over 2} − *z*
O1⋯H6	2.43	2 − *x*, {1\over 2} + *y*, {1\over 2} − *z*
Cl1⋯H11*B*	2.99	*x*, {1\over 2} − *y*, {1\over 2} + *z*
Cl3⋯H10*B*	2.96	−1 + *x*, *y*, *z*
O2⋯Cl4	3.4606 (11)	1 − *x*, −*y*, −*z*
H10*A*⋯O2	2.45	2 − *x*, −*y*, −*z*

**Table 3:** Experimental details

Crystal data	
Chemical formula	C_24_H_16_Cl_12_N_2_O_4_
*M*_r_	821.79
Crystal system, space group	Monoclinic, *P*2_1_/*c*
Temperature (K)	150
*a*, *b*, *c* (Å)	8.9549 (3), 10.5908 (4), 16.6043 (6)
β (°)	103.499 (1)
*V* (Å^3^)	1531.24 (10)
*Z*	2
Radiation type	Mo *K*α
μ (mm^−1^)	1.12
Crystal size (mm)	0.34 × 0.32 × 0.28

Data collection	
Diffractometer	Bruker APEXII CCD
Absorption correction	Multi-scan (*SADABS*; Krause *et al.*, 2015 ▸)
*T*_min_, *T*_max_	0.684, 0.736
No. of measured, independent and observed [*I* > 2σ(*I*)] reflections	12567, 3403, 3141
*R*_int_	0.023
(sin θ/λ)_max_ (Å^−1^)	0.643

Refinement	
*R*[*F* ^2^ > 2σ(*F* ^2^)], *wR*(*F* ^2^), *S*	0.021, 0.053, 1.04
No. of reflections	3403
No. of parameters	190
H-atom treatment	H-atom parameters constrained
Δρ_max_, Δρ_min_ (e Å^−3^)	0.33, −0.24

Computer programs: *APEX2* and *SAINT* (Bruker, 2007 ▸), *SHELXT* (Sheldrick, 2015*a* ▸), *SHELXL* (Sheldrick, 2015*b* ▸), *ORTEP-3 for Windows* (Farrugia, 2012 ▸) and *PLATON* (Spek, 2020 ▸).

## supplementary crystallographic information

### Crystal data

**Table:**

C_24_H_16_Cl_12_N_2_O_4_	*F*(000) = 820
*M**_r_* = 821.79	*D*_x_ = 1.782 Mg m^−^^3^
Monoclinic, *P*2_1_/*c*	Mo *K*α radiation, λ = 0.71073 Å
*a* = 8.9549 (3) Å	Cell parameters from 7701 reflections
*b* = 10.5908 (4) Å	θ = 2.3–27.2°
*c* = 16.6043 (6) Å	µ = 1.12 mm^−^^1^
β = 103.499 (1)°	*T* = 150 K
*V* = 1531.24 (10) Å^3^	Block, colourless
*Z* = 2	0.34 × 0.32 × 0.28 mm

### Data collection

**Table:**

Bruker APEXII CCD diffractometer	3141 reflections with *I* > 2σ(*I*)
φ and ω scans	*R*_int_ = 0.023
Absorption correction: multi-scan (SADABS; Krause *et al.*, 2015)	θ_max_ = 27.2°, θ_min_ = 2.3°
*T*_min_ = 0.684, *T*_max_ = 0.736	*h* = −8→11
12567 measured reflections	*k* = −13→13
3403 independent reflections	*l* = −21→21

### Refinement

**Table:**

Refinement on *F*^2^	Primary atom site location: structure-invariant direct methods
Least-squares matrix: full	Secondary atom site location: difference Fourier map
*R*[*F*^2^ > 2σ(*F*^2^)] = 0.021	Hydrogen site location: inferred from neighbouring sites
*wR*(*F*^2^) = 0.053	H-atom parameters constrained
*S* = 1.04	*w* = 1/[σ^2^(*F*_o_^2^) + (0.0231*P*)^2^ + 0.7545*P*] where *P* = (*F*_o_^2^ + 2*F*_c_^2^)/3
3403 reflections	(Δ/σ)_max_ = 0.001
190 parameters	Δρ_max_ = 0.33 e Å^−^^3^
0 restraints	Δρ_min_ = −0.24 e Å^−^^3^

### Special details

**Table:**

Geometry. All esds (except the esd in the dihedral angle between two l.s. planes) are estimated using the full covariance matrix. The cell esds are taken into account individually in the estimation of esds in distances, angles and torsion angles; correlations between esds in cell parameters are only used when they are defined by crystal symmetry. An approximate (isotropic) treatment of cell esds is used for estimating esds involving l.s. planes.

### Fractional atomic coordinates and isotropic or equivalent isotropic displacement parameters (Å^2^)

**Table:**

	*x*	*y*	*z*	*U*_iso_*/*U*_eq_	
Cl1	0.81506 (4)	0.38645 (3)	0.33748 (2)	0.02092 (8)	
Cl2	0.65657 (4)	0.08552 (3)	0.31569 (2)	0.02036 (8)	
Cl3	0.45141 (4)	0.28198 (3)	0.24334 (2)	0.01993 (8)	
Cl4	0.48797 (4)	0.05628 (3)	0.10287 (2)	0.02218 (8)	
Cl5	0.75019 (4)	0.52267 (3)	0.15453 (2)	0.02455 (9)	
Cl6	0.55737 (5)	0.31532 (4)	0.00843 (2)	0.02966 (9)	
O1	1.10843 (11)	0.32935 (10)	0.22625 (6)	0.0224 (2)	
O2	0.83761 (12)	0.05643 (10)	0.03360 (6)	0.0220 (2)	
N1	0.99701 (12)	0.19567 (11)	0.11936 (7)	0.0156 (2)	
C1	1.01234 (15)	0.25177 (13)	0.19624 (8)	0.0152 (3)	
C2	0.88902 (15)	0.19800 (12)	0.23555 (8)	0.0139 (2)	
H2	0.935586	0.153877	0.288724	0.017*	
C3	0.76483 (15)	0.29560 (12)	0.24743 (8)	0.0142 (2)	
C4	0.62421 (15)	0.20595 (12)	0.24022 (8)	0.0142 (2)	
C5	0.63197 (15)	0.15971 (12)	0.15208 (8)	0.0140 (2)	
C6	0.79769 (15)	0.10488 (12)	0.17088 (8)	0.0142 (2)	
H6	0.801142	0.017191	0.193567	0.017*	
C7	0.87382 (15)	0.11223 (12)	0.09843 (8)	0.0153 (3)	
C8	0.71121 (15)	0.36779 (12)	0.16671 (8)	0.0153 (3)	
C9	0.63396 (15)	0.28797 (13)	0.11034 (8)	0.0157 (3)	
C10	1.09720 (16)	0.21915 (14)	0.06241 (8)	0.0200 (3)	
H10A	1.112615	0.139247	0.034488	0.024*	
H10B	1.198763	0.248383	0.094445	0.024*	
C11	1.02959 (17)	0.31780 (14)	−0.00267 (8)	0.0213 (3)	
H11A	1.081293	0.311153	−0.049094	0.026*	
H11B	0.919312	0.299100	−0.024805	0.026*	
C12	1.04563 (18)	0.45260 (13)	0.03013 (8)	0.0223 (3)	
H12A	1.010849	0.455925	0.082477	0.027*	
H12B	1.155427	0.476794	0.042781	0.027*	

### Atomic displacement parameters (Å^2^)

**Table:**

	*U*^11^	*U*^22^	*U*^33^	*U*^12^	*U*^13^	*U*^23^
Cl1	0.02260 (17)	0.01966 (17)	0.01950 (16)	0.00008 (13)	0.00287 (13)	−0.00824 (12)
Cl2	0.02766 (18)	0.01698 (16)	0.01742 (15)	0.00127 (13)	0.00727 (13)	0.00505 (12)
Cl3	0.01596 (15)	0.01944 (17)	0.02574 (17)	0.00286 (12)	0.00760 (13)	−0.00038 (12)
Cl4	0.01995 (16)	0.02252 (18)	0.02291 (16)	−0.00783 (13)	0.00266 (13)	−0.00581 (13)
Cl5	0.02410 (18)	0.01230 (16)	0.0374 (2)	−0.00025 (13)	0.00750 (15)	0.00650 (13)
Cl6	0.0340 (2)	0.0339 (2)	0.01635 (16)	0.00063 (16)	−0.00359 (14)	0.00900 (14)
O1	0.0162 (5)	0.0248 (5)	0.0257 (5)	−0.0038 (4)	0.0037 (4)	−0.0082 (4)
O2	0.0253 (5)	0.0215 (5)	0.0197 (5)	−0.0021 (4)	0.0061 (4)	−0.0076 (4)
N1	0.0153 (5)	0.0151 (6)	0.0165 (5)	0.0019 (4)	0.0043 (4)	−0.0011 (4)
C1	0.0124 (6)	0.0151 (6)	0.0168 (6)	0.0048 (5)	0.0008 (5)	−0.0006 (5)
C2	0.0146 (6)	0.0121 (6)	0.0139 (6)	0.0031 (5)	0.0008 (5)	−0.0012 (5)
C3	0.0147 (6)	0.0129 (6)	0.0145 (6)	0.0014 (5)	0.0021 (5)	−0.0018 (5)
C4	0.0153 (6)	0.0118 (6)	0.0154 (6)	0.0020 (5)	0.0037 (5)	0.0016 (5)
C5	0.0144 (6)	0.0128 (6)	0.0138 (6)	−0.0016 (5)	0.0015 (5)	−0.0002 (5)
C6	0.0163 (6)	0.0116 (6)	0.0142 (6)	0.0007 (5)	0.0023 (5)	0.0003 (5)
C7	0.0163 (6)	0.0117 (6)	0.0177 (6)	0.0035 (5)	0.0036 (5)	0.0000 (5)
C8	0.0135 (6)	0.0129 (6)	0.0200 (6)	0.0024 (5)	0.0049 (5)	0.0037 (5)
C9	0.0146 (6)	0.0173 (6)	0.0146 (6)	0.0036 (5)	0.0021 (5)	0.0049 (5)
C10	0.0189 (7)	0.0217 (7)	0.0220 (7)	−0.0003 (6)	0.0100 (5)	−0.0032 (5)
C11	0.0262 (7)	0.0211 (7)	0.0172 (6)	−0.0055 (6)	0.0067 (6)	−0.0023 (5)
C12	0.0274 (7)	0.0209 (7)	0.0178 (6)	−0.0054 (6)	0.0037 (6)	−0.0022 (5)

### Geometric parameters (Å, º)

**Table:**

Cl1—C3	1.7464 (13)	C3—C4	1.5592 (18)
Cl2—C4	1.7639 (13)	C4—C5	1.5599 (17)
Cl3—C4	1.7558 (13)	C5—C9	1.5269 (18)
Cl4—C5	1.7432 (13)	C5—C6	1.5559 (18)
Cl5—C8	1.6989 (14)	C6—C7	1.5168 (18)
Cl6—C9	1.6958 (13)	C6—H6	1.0000
O1—C1	1.2098 (17)	C8—C9	1.3293 (19)
O2—C7	1.2042 (16)	C10—C11	1.523 (2)
N1—C1	1.3855 (16)	C10—H10A	0.9900
N1—C7	1.3927 (17)	C10—H10B	0.9900
N1—C10	1.4686 (17)	C11—C12	1.5228 (19)
C1—C2	1.5186 (19)	C11—H11A	0.9900
C2—C6	1.5442 (17)	C11—H11B	0.9900
C2—C3	1.5642 (17)	C12—C12^i^	1.515 (3)
C2—H2	1.0000	C12—H12A	0.9900
C3—C8	1.5203 (17)	C12—H12B	0.9900
			
C1—N1—C7	113.85 (11)	C2—C6—C5	103.10 (10)
C1—N1—C10	125.21 (11)	C7—C6—H6	111.5
C7—N1—C10	120.94 (11)	C2—C6—H6	111.5
O1—C1—N1	125.37 (13)	C5—C6—H6	111.5
O1—C1—C2	126.60 (12)	O2—C7—N1	124.52 (13)
N1—C1—C2	108.03 (11)	O2—C7—C6	127.35 (12)
C1—C2—C6	105.12 (10)	N1—C7—C6	108.13 (11)
C1—C2—C3	114.59 (11)	C9—C8—C3	107.83 (11)
C6—C2—C3	103.47 (10)	C9—C8—Cl5	128.16 (11)
C1—C2—H2	111.1	C3—C8—Cl5	124.00 (10)
C6—C2—H2	111.1	C8—C9—C5	107.78 (11)
C3—C2—H2	111.1	C8—C9—Cl6	128.08 (11)
C8—C3—C4	98.94 (10)	C5—C9—Cl6	124.06 (10)
C8—C3—C2	107.98 (10)	N1—C10—C11	111.81 (11)
C4—C3—C2	99.97 (10)	N1—C10—H10A	109.3
C8—C3—Cl1	116.29 (9)	C11—C10—H10A	109.3
C4—C3—Cl1	116.31 (9)	N1—C10—H10B	109.3
C2—C3—Cl1	115.05 (9)	C11—C10—H10B	109.3
C3—C4—C5	92.94 (9)	H10A—C10—H10B	107.9
C3—C4—Cl3	114.83 (9)	C12—C11—C10	113.62 (11)
C5—C4—Cl3	113.84 (9)	C12—C11—H11A	108.8
C3—C4—Cl2	112.95 (9)	C10—C11—H11A	108.8
C5—C4—Cl2	113.80 (9)	C12—C11—H11B	108.8
Cl3—C4—Cl2	108.08 (7)	C10—C11—H11B	108.8
C9—C5—C6	108.14 (10)	H11A—C11—H11B	107.7
C9—C5—C4	98.88 (10)	C12^i^—C12—C11	113.19 (14)
C6—C5—C4	100.27 (9)	C12^i^—C12—H12A	108.9
C9—C5—Cl4	115.59 (9)	C11—C12—H12A	108.9
C6—C5—Cl4	115.22 (9)	C12^i^—C12—H12B	108.9
C4—C5—Cl4	116.55 (9)	C11—C12—H12B	108.9
C7—C6—C2	104.81 (10)	H12A—C12—H12B	107.8
C7—C6—C5	113.96 (10)		
			
C7—N1—C1—O1	−178.12 (13)	C3—C2—C6—C5	0.55 (12)
C10—N1—C1—O1	1.4 (2)	C9—C5—C6—C7	−47.46 (14)
C7—N1—C1—C2	2.31 (14)	C4—C5—C6—C7	−150.44 (11)
C10—N1—C1—C2	−178.13 (11)	Cl4—C5—C6—C7	83.59 (12)
O1—C1—C2—C6	179.44 (13)	C9—C5—C6—C2	65.53 (12)
N1—C1—C2—C6	−1.01 (13)	C4—C5—C6—C2	−37.45 (12)
O1—C1—C2—C3	66.54 (17)	Cl4—C5—C6—C2	−163.42 (9)
N1—C1—C2—C3	−113.90 (12)	C1—N1—C7—O2	177.78 (13)
C1—C2—C3—C8	47.50 (14)	C10—N1—C7—O2	−1.8 (2)
C6—C2—C3—C8	−66.37 (12)	C1—N1—C7—C6	−2.63 (15)
C1—C2—C3—C4	150.38 (10)	C10—N1—C7—C6	177.79 (11)
C6—C2—C3—C4	36.51 (11)	C2—C6—C7—O2	−178.64 (13)
C1—C2—C3—Cl1	−84.24 (12)	C5—C6—C7—O2	−66.68 (18)
C6—C2—C3—Cl1	161.89 (9)	C2—C6—C7—N1	1.79 (13)
C8—C3—C4—C5	52.32 (10)	C5—C6—C7—N1	113.75 (12)
C2—C3—C4—C5	−57.86 (10)	C4—C3—C8—C9	−35.34 (13)
Cl1—C3—C4—C5	177.63 (9)	C2—C3—C8—C9	68.27 (13)
C8—C3—C4—Cl3	−65.69 (11)	Cl1—C3—C8—C9	−160.66 (10)
C2—C3—C4—Cl3	−175.87 (8)	C4—C3—C8—Cl5	145.71 (10)
Cl1—C3—C4—Cl3	59.62 (12)	C2—C3—C8—Cl5	−110.68 (11)
C8—C3—C4—Cl2	169.74 (9)	Cl1—C3—C8—Cl5	20.39 (15)
C2—C3—C4—Cl2	59.57 (11)	C3—C8—C9—C5	0.64 (14)
Cl1—C3—C4—Cl2	−64.95 (11)	Cl5—C8—C9—C5	179.54 (10)
C3—C4—C5—C9	−51.85 (10)	C3—C8—C9—Cl6	−176.19 (10)
Cl3—C4—C5—C9	66.99 (11)	Cl5—C8—C9—Cl6	2.7 (2)
Cl2—C4—C5—C9	−168.55 (9)	C6—C5—C9—C8	−69.69 (13)
C3—C4—C5—C6	58.55 (10)	C4—C5—C9—C8	34.26 (13)
Cl3—C4—C5—C6	177.40 (9)	Cl4—C5—C9—C8	159.47 (10)
Cl2—C4—C5—C6	−58.15 (11)	C6—C5—C9—Cl6	107.30 (11)
C3—C4—C5—Cl4	−176.38 (9)	C4—C5—C9—Cl6	−148.75 (10)
Cl3—C4—C5—Cl4	−57.54 (12)	Cl4—C5—C9—Cl6	−23.54 (15)
Cl2—C4—C5—Cl4	66.92 (11)	C1—N1—C10—C11	−96.26 (15)
C1—C2—C6—C7	−0.47 (13)	C7—N1—C10—C11	83.27 (15)
C3—C2—C6—C7	120.07 (11)	N1—C10—C11—C12	76.43 (15)
C1—C2—C6—C5	−119.99 (10)	C10—C11—C12—C12^i^	−169.86 (15)

Symmetry code: (i) −*x*+2, −*y*+1, −*z*.

### Hydrogen-bond geometry (Å, º)

*Cg*1 is the centroid of the N1/C1/C2/C6/C7 pyrrolidine ring.

**Table:**

*D*—H···*A*	*D*—H	H···*A*	*D*···*A*	*D*—H···*A*
C6—H6···O1^ii^	1.00	2.43	3.3867 (16)	161
C10—H10*A*···O2^iii^	0.99	2.45	3.4402 (17)	178
C12—H12*B*···Cl2^iv^	0.99	2.80	3.5299 (15)	131
C3—Cl1···*Cg*1^iv^	1.75 (1)	3.89 (1)	4.9389 (14)	117 (1)

Symmetry codes: (ii) −*x*+2, *y*−1/2, −*z*+1/2; (iii) −*x*+2, −*y*, −*z*; (iv) −*x*+2, *y*+1/2, −*z*+1/2.
